# Adrenergic Myocarditis in Pheochromocytoma

**DOI:** 10.1186/1532-429X-13-4

**Published:** 2011-01-11

**Authors:** Alberto Roghi, Patrizia Pedrotti, Angela Milazzo, Edgardo Bonacina, Chiara Bucciarelli-Ducci

**Affiliations:** 1 Non-invasive Cardiac Imaging Laboratory, CMR Unit, Department of Cardiology and Cardiovascular Surgery, Niguarda Ca'Granda Hospital, Milan, Italy; 2 Pathology Laboratories, Niguarda Ca'Granda Hospital, Milan, Italy; 3 Bristol Heart Institute, NIHR Cardiovascular Biomedical Research Unit, University Hospitals Bristol Foundation Trust, Bristol, UK; 4 Royal Brompton Hospital Foundation Trust, NIHR Cardiovascular Biomedical Research Unit, Imperial College, London, UK

## Abstract

The clinical presentation of pheochromocytoma is variable and many biochemical and imaging methods have been suggested to improve the diagnostic accuracy of what has been termed "the great masquerader". This case-report is of a middle-aged woman with a non-specific clinical presentation suggesting acute coronary syndrome or subacute myocarditis. Cardiovascular magnetic resonance (CMR) at presentation showed myocardial edema and intramyocardial late gadolinium enhancement (LGE). An adrenal mass was seen, which was confirmed as pheochromocytoma and surgically removed. Our case shows evidence for acute adrenergic myocarditis, with resolution of both the edema and the LGE after surgical excision.

## Background

Pheochromocytomas are cathecolamine-producing tumors derived from the sympathetic or parasympathetic nervous system. The clinical presentation is variable, ranging from adrenal incidentalomas to patients with hypertensive crisis or, rarely, with congestive heart failure. The high circulating levels of catecholamines resulting from a pheochromocytoma may cause direct myocardial injury. Focal myocardial necrosis and inflammatory cell are present in 50% of patients who die with pheochromocytoma and may contribute to clinically significant left ventricular failure. The diagnosis is based on documentation of catecholamine excess by biochemical testing and localization of the tumor by imaging.

## Case Presentation

**Figure 1:**
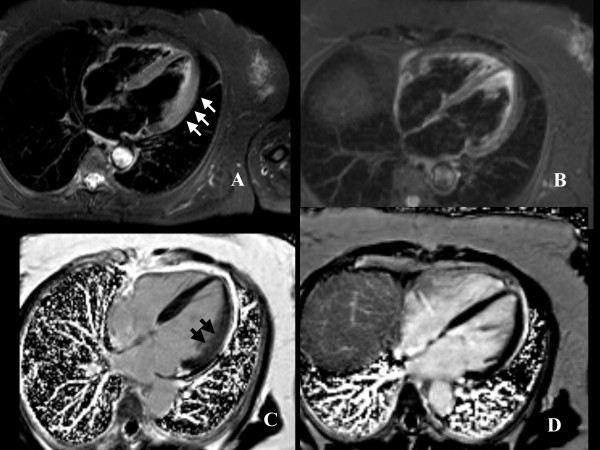
**STIR and LGE images.** A, four chamber view, T2-weighted image showing thickening of lateral wall with edema (white arrows); B, post-surgical follow-up T2-weighted images showing normalization of wall thickness and regression of edema; C, post-contrast images showing diffuse myocardial enhancement (black arrows); D, post-surgical follow-up post-contrast images showing regression of myocardial enhancement.

A 64-year-old woman was referred to our hospital with symptoms of breathlessness, dizziness and palpitations. Her past medical history was relevant for hypertensive episodes and for a recent episode of gastroenteritis. Clinical examination of the heart, lungs and abdomen was unremarkable. The ECG showed left ventricular (LV) hypertrophy with ST-segment depression and her blood pressure was 180/110 mmHg. Chest X-ray was normal. Troponin I values was in the normal range, Creatine-kinase (CK-MB) was slightly increased (5.5 U/L, normal values 0.0-5.0 U/L) as the leukocyte count and C-reactive protein values (0.6 mg/dL, normal values 0.0-0.5 mg/dL). Echocardiogram showed mild left ventricular (LV) increased wall thickness and hypokinesia of postero-lateral segments, but normal overall systolic function (LVEF 56%). Based on the echocardiographic findings and clinical presentation, the final diagnosis was unclear but sub-acute myocarditis and acute coronary syndrome with subendocardial ischemia were considered the two most likely differential diagnosis. The patient was subsequently referred for a cardiovascular magnetic resonance (CMR) study to further evaluate LV function and myocardial tissue characterization. The LV cine images confirmed the abnormalities observed with echocardiography (Additional file 1). Non invasive myocardial tissue characterization with T2-weigthed imaging demonstrated myocardial edema of the postero-lateral segments, whereas the T1-weighted late gadolinium enhancement (LGE) images showed diffuse and patchy myocardial enhancement, both consistent with acute non-ischemic myocardial damage (Figure 1A, C). In particular, the CMR features of increased wall thickness, hypokinesia, myocardial edema and patchy pattern of LGE were pathognomonic of acute myocarditis and excluded ischemic heart disease.

**Figure 2:**
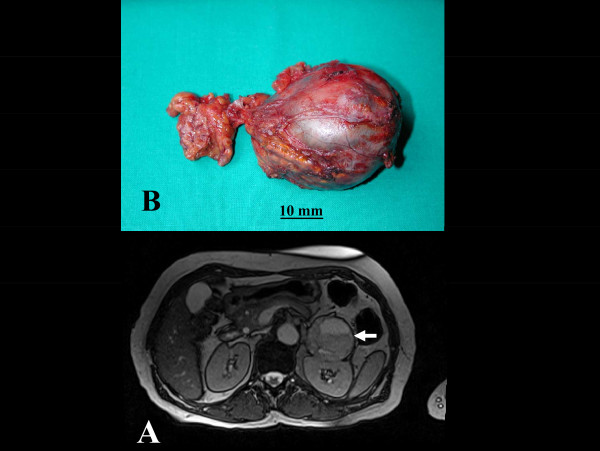
**Pre-surgical MR and macroscopic anatomy images.** A, abdomen MRI showing large left surrenal mass (white arrow); B, pheochromocytoma after surgical resection.

**Figure 3:**
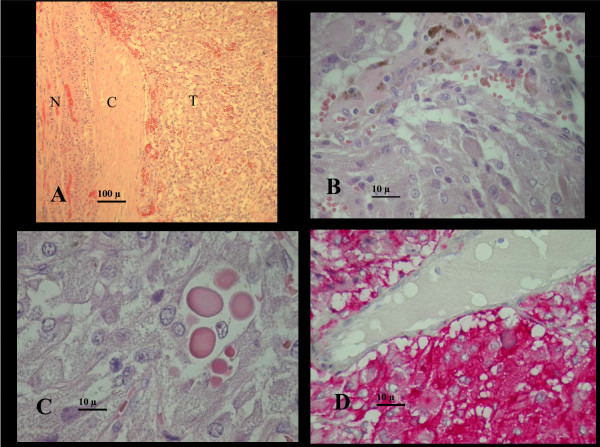
**Histology data.** A, low magnification showing the watershed between tumor (T) and normal gland (N), and the tumoral "pseudocapsule"(C). Organoid ( alveolar) arrangement of tumor cells with nests or cords of poligonal eosinophilic tumor cells in a highly vascularised stroma.B, tumor cells showing finely granular, pinkish-brown, delicate cytoplasm, ovoid nuclei with small regular nucleoli. Haemosiderin from small intratumor haemorrages is shown at the top. C, hyaline globules are present in 47% of pheochromocytomas: they are PAS positive, diastase resistant eosinophilic rounded masses of degenerated cytoplasmatic organules. D, synaptophysin immunostain showing intense cytoplasmatic positivity of tumor cells (red), a venule is shown on the top for negative comparison.

In addition, a large incidental mass was identified in the left suprarenal gland, suggesting pheochromocytoma (Figure 1b). Only about 5% of adrenal incidentalomas, usually detected by CT or MRI, prove to be pheochromocytomas after endocrinologic evaluation. On the basis of the clinical presentation and of CMR findings, endocrinologic workup was carried out. Urinary cathecolamines and methanephrines were evaluated and found to be increased: urinary adrenaline 163 ng/24 h (normal values 2-22 ng/24 h), urinary noradrenaline 517 ng/24 h (normal values 12-85 ng/24 h), urinary metanephrine 3387 ng/24 h (normal values 74-297 ng/24 h), urinary normetanephrine 4085 ng/24 h (normal values 105-354 ng/24 h). The diagnosis of pheochromocytoma was therefore confirmed and the patient underwent surgical resection of the adrenal mass (Figure 2B). The histological examination (Figure 3) confirmed the diagnosis of secreting pheochromocytoma. The patient was then discharged home. Three months later she underwent a follow-up CMR, showing normalization of wall thickness (Additional file 2), regression of both myocardial edema and LGE (Figure 1B, D).

## Discussion

Pheochromocytoma is a catecholamine-secreting tumor that arises mainly (80% of the cases) from the adrenal medulla or from extra-adrenal abdominal paraganglion tissue [1]. The prevalence of pheochromocytoma in patients with hypertension is 0.1-0.6%, and secretes both epinephrine and norepinephrine in at least 50% of cases.

A diagnosis of pheochromocytoma should be considered in patients with hypertension and unexplained symptoms. The classic presentation with severe headache, palpitations, chest pain, sweating, tremor is the most common but epinephrine and dopamine-secreting tumors also occur and patients with these tumors present with hypotension or cardiogenic shock when beta adrenergic stimulation overrides alpha-adrenergic stimulation. High catecholamine level can cause direct myocardial damage with focal degeneration and contraction band necrosis of the myocytes, monocytic infiltration, medial thickening of small and medium size coronary arteries and interstitial fibrosis. The clinical picture of catecholamine myocarditis is common in autopsies studies of patients died from pheochromocytoma as well as those died from the stress of physical assault [2-4].

CMR is a non-invasive imaging technique that can diagnose acute and chronic myocarditis with combined T2- and T1-weighted after contrast (LGE) that highlight the presence of acute myocardial damage with myocardial inflammation and edema [5]. In our case, a follow-up scan proved to be helpful in confirming the reversible nature of the myocardial damage observed in the first scan. The role of serial scanning in patients with acute myocarditis is uncertain based on the limited current literature.

## Conclusion

For our patient, comprehensive CMR showed reversible acute adrenergic toxicity in addition to identifying the pheochromocytoma.

## Consent Statement

Written informed consent was obtained from the patient for publication of this case report and accompanying images. A copy of the written consent is available for review by the Editor-in-Chief of this journal.

## Competing interests

The authors declare that they have no competing interests.

## Authors' contributions

AR conceived the study and drafted the manuscript, PP carried out the CMR examinations, AM participated to the collection and presentation of clinical and images data, EB carried out the histological examination and the histology images, CBD participated to the revision of the manuscript. All authors read and approved the final manuscript.

## Supplementary Material

Additional file 1 **Four chambers cine at admission**. CMR four-chamber cine showing increased LV wall thickness with normal function Click here for file

Additional file 2 **Four chambers cine at post-surgical follow-up**. CMR four-chamber cine showing normalization of LV wall thickness Click here for file
